# Future directions for HIV service delivery research: Research gaps identified through WHO guideline development

**DOI:** 10.1371/journal.pmed.1003812

**Published:** 2021-09-23

**Authors:** Nathan Ford, Ingrid Eshun-Wilson, Wole Ameyan, Morkor Newman, Lara Vojnov, Meg Doherty, Elvin Geng

**Affiliations:** 1 Department of HIV, Viral Hepatitis and STIs, World Health Organization, Geneva, Switzerland; 2 Division of Infectious Diseases, Washington University School of Medicine, St. Louis, Missouri, United States of America

## Abstract

Nathan Ford and co-authors discuss the systematic identification of research gaps in improving HIV service delivery.

**Summary points**

- Improvements in HIV service delivery are key to bringing countries closer to achieving the target of ending AIDS as a public health threat and situating HIV treatment and care as part of universal healthcare coverage.
- The World Health Organization (WHO) guideline development process is recognized as one approach to identifying research gaps. Systematic reviews form the basis of recommendations formulated by an expert guideline development group, which is also tasked to identify research gaps.
- The 2021 WHO HIV Service Delivery Guideline process identified 27 research gaps grouped into 8 areas where more research is needed to support enhancement and implementation of the new recommendations across the cascade of care.
- Areas covered by the WHO Service Delivery Guideline include antiretroviral therapy (ART) initiation outside the health facility, frequency of visits/refills, tracing and reengagement in care, assessing adherence, integration of HIV and sexual and reproductive health services, integration of HIV and diabetes and hypertension care, psychosocial interventions for adolescents, and task sharing of specimen collection and point-of-care testing.
- Key areas identified by the guideline process that could benefit from future research include tools to support ART initiation outside the health facility, outcomes of spacing of clinical visits/drug refills beyond 6 months, tailored support to minimize disengagement and support reengagement along the continuum of care, and accurate, feasible measures of adherence.
- Strategies of integration of HIV and sexual and reproductive health services and hypertension and diabetes care, costs and cost-effectiveness of psychological support interventions, the performance of newer point-of-care technologies by nonlaboratory personnel, and the impact of diagnostic integration across disease types were also identified as key areas that would benefit from future research.

## Introduction

In 2016, the World Health Organization (WHO) published guidelines recommending that all people living with HIV start antiretroviral therapy (ART) irrespective of disease status. This recommendation has been widely adopted by countries, substantially increasing the number of individuals eligible to start treatment [1]. These 2016 guidelines also provide several recommendations promoting differentiated service delivery as a way to simplify and rationalize care delivery by reducing requirements to visit health services; recommendations supporting task sharing, decentralization, and service integration were made with the aim of increasing access to care while maintaining quality [2].

Since the publication of these guidelines, several important studies have provided additional evidence to help improve ART start. In particular, accelerating the offer of ART, including on the same day that a diagnosis is made, has been found to increase uptake of ART and viral suppression [3,4]. It was recognized that an important number of patients continued to present with advanced HIV disease, either because they were diagnosed late in their infection or because they had disengaged from care and stopped taking treatment [5]. The results of 2 trials supported the provision of a package of care including diagnostics or prophylactics to all patients presenting with advanced HIV disease to reduce mortality [6]. Recommendations supporting rapid initiation of ART and provision of a package of care for advanced HIV disease were included in a subsequent WHO guideline published in 2017 [7].

At the end of 2018, WHO convened an expert consultation to define future priorities for HIV treatment service delivery [8]. The outcomes of this consultation provided a roadmap for the updated HIV service delivery guidelines that were published in April 2021 [9]. The new guidelines put forward recommendations on starting treatment outside of the health facility, the frequency of clinic visits and drug dispensing, tracing and reengagement in care, assessing adherence, integration of HIV and sexual and reproductive health and noncommunicable disease services, task sharing of specimen collection and point-of-care testing, and psychosocial support for adolescents and young adults.

Systematic reviews provide the basis for the formulation of guideline recommendations and also provide an opportunity to identify important evidence gaps to be addressed in future guidelines. As part of a collection of articles addressing evidence to inform policy and practice of HIV service delivery [8], this article summarizes the research gaps that were identified during the development of these guidelines.

## Identifying research gaps as part of guideline development

The WHO guideline development process is recognized as one approach to identifying research gaps [10]. A guideline development group is convened that includes a diversity of experts and relevant stakeholders from across all WHO regions affected by the public health problem. Systematic reviews form the basis of formulating recommendations through an assessment of potential benefits and harms, values and preferences, feasibility and resource use regarding the recommended intervention [11]. Each of the areas addressed by the updated WHO HIV Service Delivery Guidelines was informed by 1 or more systematic reviews. The Grading of Recommendations, Assessment, Development and Evaluations (GRADE) framework is used to determine the certainty of the evidence, which is a key driver of the strength of the recommendation [12]. Five domains—risk of bias, reporting bias, imprecision, indirectness, and inconsistency—are used to rate the certainty of the evidence. These domains reflect limitations in the existing evidence, either because the evidence base is small, or comprised of studies with contradictory findings, or studies that have important methodological flaws. For those interventions supported by low or moderate certainty evidence, additional research could increase the certainty of the evidence and the strength of the recommendation [13].

Guideline development groups are also tasked to formulate research gaps considering the limitations of the evidence identified by the systematic reviews. Once drafted, the guideline is reviewed by an external review group, which again is comprised of leading experts and relevant stakeholders from across all WHO regions; this group can provide further inputs into the research gaps that have been identified. Three types of evidence gaps are commonly identified through this process. First, there is insufficient evidence to inform which interventions should be recommended to address a given service delivery challenge (e.g., the most accurate way to measure medication adherence); second, evidence exists but is insufficient to support a recommendation for a specific population group (e.g., pregnant women); third, there is no evidence from a particular setting (e.g., community settings) or geographical regions (e.g., Latin America), limiting the scope of implementation of a given recommendation.

The guideline development group for the WHO 2021 HIV Service Delivery Guidelines [14] included 19 experts from 13 countries who appraised the evidence derived from 17 systematic reviews, in addition to surveys on values and preferences of health workers and recipients of care. The draft guidelines, including a draft set of research gaps, were reviewed by an external review group comprising 13 experts from 10 countries. Through this process, 27 research gaps were identified, grouped into 8 areas where more research is needed to support enhancement and implementation of the new recommendations. These are summarized in Table 1 and detailed below per recommendation area.

**Table 1:** Research gaps identified during the HIV service delivery guideline development process.

Area of intervention	Research gaps	Evidence
ART initiation outside the health facility	Client preferences about where to start ART and how to link to care by age, population, and setting	Mixed methods
	Tools to support initiation outside the health facility	Quantitative (comparative intervention)
	Optimum staffing complement and minimum set of skills	Health service research
	Effect on household spending and catastrophic costs	Economic evaluation
	High-quality observational research during implementation to evaluate short and long-term outcomes	Quantitative
Frequency of visits/refills	Outcomes of spacing of clinical visits/drug refills >6 months	Quantitative comparative intervention)
Tracing and reengagement in care	Tailored support to minimize disengagement and support reengagement along the continuum of care	Quantitative (comparative intervention)
	Acceptability and effectiveness of approaches for tracing and reengagement by population	Mixed methods
Assessing adherence	Accurate, feasible measures of adherence	Diagnostic accuracy
Integration of HIV and sexual and reproductive health services	Approaches to integration that lead to better uptake of sexual and reproductive health services, including contraception	Health service research
	Strategies of integration in different health systems and social contexts	Health service research
	Provision of contraception in the context of less frequent clinical and ART refill visits	Quantitative
Integration of HIV and diabetes and hypertension care	Long-term data on the health outcomes of people living with HIV who have noncommunicable diseases	Longitudinal cohort
	Cost-effectiveness of various models of integrated care	Economic evaluation
	Supply chain optimization	Health service research
	Health promotion to encourage lifestyle changes among people living with HIV	Quantitative (comparative intervention)
	Integration of hypertension and diabetes care with common differentiated models of service delivery	Implementation research
	Values and preferences related to care delivery	Qualitative
Psychosocial interventions for adolescents and young adults	Interventions that improve outcomes for different groups of adolescents and young adults living	Quantitative (comparative intervention)
	Content and delivery strategies for interventions to involve parents and caregivers	Health service research
	Training, supervision, and support for facilitators of psychosocial interventions	Health service research
	Costs and cost-effectiveness of psychological support interventions	Economic evaluation
	Long-term outcomes of psychosocial interventions	Longitudinal cohort
Task sharing of specimen collection and point-of-care testing	Performance of newer point-of-care technologies by nonlaboratory personnel	Diagnostic accuracy
Balanced integration of diagnostic services	Impact of diagnostic integration across disease types	Quantitative (comparative intervention)
	Best practices for diagnostic integration	Implementation research
	Quality assurance approaches	Implementation research

ART, antiretroviral therapy.

## ART initiation outside the health facility

### Background

Community-based HIV testing approaches are a key component of any HIV testing strategy [15]. Rapid initiation of ART, including starting on the same day of a positive diagnosis, has also been widely adopted in policy and practice [6]. However, there are substantial losses to care between community HIV testing and ART initiation, with proportions linking to care as low as 14% for home-based testing and 10% for community-based testing [16]. A study from South Africa and Zambia found that people testing positive in the community often delayed starting ART because of issues related to the quality of care and stigma associated with accessing care [17]. Other cited concerns include lack of time [18] and concern about long clinic waiting times [19].

### What was found

The recommendation to provide out of facility ART initiation was supported by evidence from 3 randomized trials and 4 observational studies carried out in sub-Saharan Africa, except for 1 study that was carried out in Haiti [20]. Offering ART initiation outside the health facility was associated with an increase in the proportion of people starting ART, increased retention in care at 6 to 12 months following ART initiation, and increased viral load suppression at 12 months. Only 1 study was conducted in adolescents, and overall the certainty of the evidence was rated as low due to substantial heterogeneity in terms of effects, strategies, and populations.

### Future research

Research is needed to improve understanding of client preferences about where to start ART and how to link to and differentiate care across different age groups, populations, and settings. Tools to support initiation outside the health facility need to be identified and evaluated. The identified studies showed substantial variability in the size of the community treatment teams, and implementation research would be valuable in defining the optimum staffing complement and minimum set of skills required. In addition, high-quality observational data collected during implementation will help with early identification of challenges that may arise and be addressed when community ART initiation is implemented outside of more controlled research environments. Finally, evidence is needed on how ART initiation outside the facility affects household spending.

## Frequency of visits/refills

### Background

In 2016, WHO recommended clinical visits every 3 to 6 months and dispensing ART lasting 3 to 6 months for people established on ART [7]. Two distinct recommendations were made to underscore the point that clinical visits and medication dispensing should be considered separately. These recommendations have been broadly adopted by national policy, but there is uncertainty about the optimal visit/dispensing frequency.

### What was found

The systematic review supporting the 2021 guidelines found 23 studies, including 8 randomized trials. Except for 1 study done in Haiti, all studies were conducted in sub-Saharan Africa. Overall, there was no difference in retention in care or viral suppression comparing 3- and 6-month visit frequency or ART dispensing. There was insufficient evidence to support extending the frequency of visits or dispensing beyond this period. Nevertheless, there are contexts in which annual clinical visits are the standard of care and may benefit clients and reduce costs for health systems and outcomes associated with this approach should be assessed. Most of the studies to date have been conducted among adults, and more evidence is needed for children and adolescents. Evidence from a greater diversity of geographical settings would also be valuable.

### Future research

Given the range of differentiated service delivery models being considered by national HIV treatment programs (e.g., community- and facility-based adherence clubs), the guideline development group considered that research on the comparative effectiveness, comparative cost-effectiveness, and comparative implementability of these models would be important to inform policy and prioritization. The introduction of one model to existing models would be useful to understand the additional contribution of a program to “mosaic effectiveness” [21].

## Tracing and reengagement in care

### Background

Losses to care remain substantial in all regions and are especially high in southern Africa, affecting all age groups [22–24]. People are known to disengage from care for a variety of reasons, and successful tracing and reengagement allows people to reinitiate ART and receive the care they need to prevent deterioration of health status and achieve viral suppression.

### What was found

The systematic review found 37 studies that assessed activities to trace individuals who have disengaged and identified interventions to support reengagement. These studies were carried out in Africa, the United States of America, and Australia; 8 studies included children and adolescents. Approaches to tracing varied between low- and high-income countries and included remote communication (phone, text, mail, and email), in-person tracing, and a combination of approaches. Overall, the review found that, when asked, 58% (95% CI 51 to 65%) were willing to reengage in care. Nine studies compared reengagement interventions to a control condition, and, in this analysis, reengagement interventions improved return to care compared to standard care.

### Future research

Several studies have described the most common reasons for disengagement from care either before or after initiating ART [25–28]. Research is needed to tailor support that responds to these drivers to minimize disengagement and support reengagement at different stages along the continuum of care. Qualitative research is important to understand the most acceptable and effective methods of tracing and reengagement. Future research should include disaggregation of approaches based on the population group (e.g., key populations), sex, and age and investigate approaches to facilitate return and support retention after return.

A recent systematic review of published criteria and definitions across the HIV care cascade in sub-Saharan Africa found that proportions of individuals retained in care at each step of the HIV care cascade cannot be compared between studies due to the highly variable definitions used for numerators and denominators; this review concluding that there is a need for standardized methods and definitions [29]. In general, the review process of reengagement also revealed an urgent need to standardize conceptualization and representation of the reengagement cascade and how to harmonize quantification of effects. For example, when seeking to assess a descriptive estimate of the incidence of “return” among lost patients, some studies reported return among all patients who had a missed visit in the electronic medical record, while other studies examined return in those who the facility tried to contact or among those successfully contacted [30]. In addition, the amount of time elapsed between the missed visit and when studies began to consider patients to be lost and therefore “count” their subsequent visits as “returns to clinic,” varied from a few days to several weeks or even months, while in other studies, this interval was not specified [31–33], again yielding estimates with fundamentally different significance. Consistent nomenclature, harmonized metrics, and a transparent description of the reengagement cascade would help to advance the science.

## Assessing adherence

### Background

WHO strongly recommends that adherence support interventions should be provided to people on ART [7]. Viral load monitoring is the gold standard for monitoring adherence and confirming treatment response, but knowledge of adherence is important to support decisions about whether a recipient of care is eligible for simplified models of service delivery and whether to switch treatment regimens when viral load is unsuppressed. Simple, affordable measures suggested by WHO to measure adherence include pill counts, pharmacy refill records, and self-reporting [34].

### What was found

A systematic review identified 50 studies to assess the comparative diagnostic accuracy of different adherence measures, including pill counts, pharmacy refill records, and self-reporting [35]. Overall, the review found that all adherence measures had low sensitivity for identifying people who are nonadherent and have unsuppressed viral loads.

### Future research

Composite adherence measures, such as combining self-report with pharmacy refill or tablet count, appeared to have higher sensitivity. Research is encouraged to identify the most accurate measures of adherence—alone or in combination—that are feasible in settings with limited resources as a complement to viral load testing. Specific population groups face additional challenges to adherence, and these should be considered.

## Integration of HIV and sexual and reproductive health services

### Background

Integration of services for different diseases that commonly affect the same person can lead to improved uptake and outcomes associated with these services. In 2019, an estimated 1.1 billion women of reproductive age needed family planning, and, of these, 270 million had an unmet need for contraception. Across regions, sex workers have a greater unmet need for contraception than the general population, and there are reports of excessive reliance on using condoms alone instead of the recommended dual protection [36–39]. WHO emphasizes the importance of linking sexual and reproductive health and rights and HIV for adolescent girls and young women [40].

### What was found

A systematic review published in 2019 found that the proportion of women receiving an HIV test during the study period ranged from 35% to 99% for integrated services and from 20% to 95% for nonintegrated services or services integrated at a lower level [41]. The proportion of women accessing HIV services using contraception ranged from 54% to 80% for integrated services and from 10% to 83% for nonintegrated services [41]. The review included 6 studies—1 cluster randomized trial carried out in Uganda and 5 nonrandomized cluster trials carried out in Kenya, Eswatini, and the USA. The overall certainty of evidence for all outcomes was very low. Another systematic review of 14 studies found that integrating family planning into HIV care and treatment settings was associated with higher levels of use and knowledge of modern methods of contraception among women living with HIV [42].

### Future research

Research is encouraged to identify approaches to integration that lead to better uptake of sexual and reproductive health services, including contraception; such research should also consider integrating cervical cancer screening and vaccination. Implementation research is encouraged to evaluate different strategies of integration in different health systems and social contexts, including providing contraception in the context of less frequent clinical and ART refill visits.

## Integration of HIV and diabetes and hypertension care

### Background

According to WHO, 15 million people 30 to 69 years old die prematurely from noncommunicable diseases every year, and 85% of these people live in low- and middle-income countries. Diabetes and hypertension are the major cardiovascular risk factors for target organ damage of the brain, heart, and kidneys. An estimated 425 million people in low- and middle-income countries currently have diabetes. The prevalence of hypertension among adults in low- and middle-income countries is estimated to exceed 20% [43]. With continued adherence to treatment, people living with HIV can expect a near-normal life expectancy [44] and an increased risk of noncommunicable diseases (especially cardiovascular diseases, cervical cancer, depression, and diabetes) related to HIV itself and to ART-related side effects [45,46].

### What was found

The systematic review identified 2 interrupted time series studies [47,48] and 3 cluster randomized trials [49–51]; 4 studies were carried out in sub-Saharan Africa, and 1 was carried out in India. These studies found that integrated models of care that include hypertension or diabetes or multidisease approaches may increase the number of people controlling both blood pressure and HIV. The overall certainty of evidence was very low, and the available evidence is limited [52].

### Future research

The following research gaps were identified: long-term data on the health outcomes of people living with HIV who have noncommunicable diseases, cost-effectiveness data for various models of integrated care, and implementation research on optimizing the supply chain. Research can help to define health promotion activities that encourage lifestyle changes and protect against noncommunicable diseases among people living with HIV who may face stigma and other challenges to receiving health promotion through the usual channels. Research is also needed to inform how hypertension and diabetes care can be integrated with the common differentiated models of HIV service delivery. Qualitative research can inform the values and preferences of people living with HIV and noncommunicable diseases related to how care is delivered.

## Psychosocial interventions for adolescents and young adults

### Background

Adolescents and young adults living with HIV face distinct challenges as they navigate the healthcare system, take on responsibilities of managing their own care and treatment, and confront issues relating to stigma and disclosure [53]. These challenges include numerous mental and social issues, including depression, stigma, isolation, difficulties with treatment adherence and retention, sexual risk-taking practices, and substance use [54].

### What was found

A systematic review identified 30 randomized controlled trials of psychosocial interventions for adolescents and young adults [55]; most studies were carried out in the USA (18 studies) or sub-Saharan Africa (9 studies). Psychosocial interventions improved adherence to ART and increased the proportion of individuals with viral suppression.

### Future research

Additional research is required to identify interventions that improve outcomes for different groups of adolescents and young adults living with HIV, including those with disabilities, mental health conditions, those who acquired HIV perinatally versus horizontally, younger adolescents, those out of school, orphans, ethnic minority groups, key populations, those who are pregnant, and those living in contexts of adversity such as extreme poverty and humanitarian emergencies. Research is also needed to assess the effectiveness of delivery strategies that involve parents and caregivers for both younger and older adolescents.

Further research is needed to inform feasible and effective training, supervision, and implementation of support models at scale for facilitators of psychosocial interventions, including peer providers. Intervention studies should include methods to report costs. Studies are encouraged to use standardized outcome definitions to enhance the comparability of findings. Lastly, follow-up beyond the immediate postintervention period is needed to understand the long-term impact of psychosocial interventions.

## Task sharing of specimen collection and point-of-care testing

### Background

Access to diagnostic testing and sample collection remains low in many resource-limited settings, partly because of shortages of human resources, especially in rural settings. The lack of skilled laboratory professionals at healthcare facilities may require the involvement of lower cadres of healthcare workers.

### What was found

The systematic review identified 65 studies that assessed the diagnostic accuracy of point-of-care testing when performed by nonlaboratory personnel [56]. Most studies (86%) were carried out in Africa. The certainty of the evidence was rated as moderate. Diagnostic accuracy was found to be similar for point-of-care CD4, early infant diagnosis, cryptococcal antigen lateral flow assays, syphilis testing, and several laboratory markers when these tests were performed by nonlaboratory personnel. For viral load testing, point-of-care viral load testing reduced turnaround time of results to clinicians.

### Future research

Additional diagnostic accuracy studies directly comparing the performance of newer point-of-care technologies infant diagnosis and viral load testing between nonlaboratory personnel and laboratory professionals would be valuable.

## Discussion

The HIV response has progressively evolved with clear direction coming from the science of HIV service delivery.

This article summarizes research questions that were established through a WHO guideline development process to make a series of recommendations to improve HIV service delivery in 2021.

The identification of research questions through the guideline development process has the advantage of being informed by updated systematic reviews of the subject area including an assessment of the certainty of the evidence through the GRADE process and interpretation by a diverse group of experts representing all WHO regions, including implementers and end users. Such an approach is restricted to the particular challenges addressed by the guideline. Other related guidelines developed by WHO in 2021 include screening and treatment to prevent cervical cancer [57] and the management of sexually transmitted infections [58]; these guidelines include research gaps to improve the management of these challenges. Another limitation to note is that research questions identified through guideline development reflect the views of those individuals who comprised the guideline development group and cannot be taken to represent the full spectrum of research gaps that exist within HIV service delivery research. Other approaches have been used by WHO to identify research priorities include expert consultation alone, literature reviews, multicriterion decision analysis, interviews, surveys, Child Health and Nutrition Research Initiative (CHNRI), Delphi, and Council on Health Research for Development (COHRED) 3D [59]. It can be anticipated that the application of 1 or more of these approaches would have identified different research priorities (such as the impact of pharmacy stockouts on adherence and viral suppression).

During the initial response to the Coronavirus Disease 2019 (COVID-19) pandemic, a number of rapid adaptions to service delivery were made to ensure continuity of essential health services [60]. It will be important to ensure that beneficial health service adaptions implemented in response to an emergency situation and are known to improve outcomes—for example, extended ART refills and community dispensing—are formally integrated into routine care, and the effects of novel service delivery adaptions are evaluated.

While evidence from randomized and observational study designs has made an important contribution—answering questions related to, for example, task sharing [61] and decentralization of ART initiation [62]—implementation science is playing an increasingly important role, helping answer questions about why effective interventions are not reaching the people who could benefit from them and how health system failures can prevent the scale up of quality services [63].

As service delivery evolves, it is important to ensure that improvements align with the lives of recipients of care to decrease the direct and indirect costs associated with seeking and receiving healthcare. A person-centered approach to delivering HIV services will be critical to bringing countries closer to achieving the target of ending AIDS as a public health threat and situating HIV treatment and care as part of universal healthcare coverage.

## Abbreviations

ART: antiretroviral therapy
CHNRI: Child Health and Nutrition Research Initiative
COHRED: Council on Health Research for Development
COVID-19: Coronavirus Disease 2019
GRADE: Grading of Recommendations, Assessment, Development and Evaluations
WHO: World Health Organization
